# Moses and Moses 2.0 for Laser Lithotripsy: Expectations vs. Reality

**DOI:** 10.3390/jcm11164828

**Published:** 2022-08-18

**Authors:** Mariela Corrales, Alba Sierra, Olivier Traxer

**Affiliations:** 1 GRC Urolithiasis No. 20 Tenon Hospital, Sorbonne University, F-75020 Paris, France; 2 Department of Urology AP-HP, Tenon Hospital, Sorbonne University, F-75020 Paris, France

**Keywords:** holmium, laser, Moses, Lumenis, kidney, ureter, lithotripsy, endourology, stones, lithiasis

## Abstract

Moses technology was born with the aim of controlling the Moses effect present in every single Ho:YAG laser lithotripsy. The capacity to divide the energy pulse into two sub-pulses gained popularity due to the fact that most of the energy would be delivered in the second pulse. However, is this pulse modulation technique really better for endocorporeal laser lithoripsy? A review of the literature was performed and all relevant clinical trials of Moses 1.0 and 2.0, as well as the lab studies of Moses 2.0 carried out up to June 2022 were selected. The search came back with 11 clinical experiences (10 full-text clinical trials and one peer-reviewed abstract) with Moses 1.0 and Moses 2.0, and three laboratory studies (peer-reviewed abstracts) with Moses 2.0 only. The clinical experiences confirmed that the MT (1.0) has a shorter lasing time but lower laser efficacy, because it consumes more J/mm^3^ when compared with the LP Ho:YAG laser (35 W). This gain in lasing time did not provide enough savings for the medical center. Additionally, in most comparative studies of MT (1.0) vs. the regular mode of the HP Ho:YAG laser, the MT did not have a significant different lasing time, operative time or stone-free rate. Clinical trials with Moses 2.0 are lacking. From what has been published until now, the use of higher frequencies (up to 120 Hz) consumes more total energy and J/mm^3^ than Moses 1.0 for similar stone-free rates. Given the current evidence that we have, there are no high-quality studies that support the use of HP Ho:YAG lasers with MT over other lasers, such as LP Ho:YAG lasers or TFL lasers.

## 1. Introduction

After 30 years, holmium:yttrium-aluminum-garnet (Ho:YAG) remains the gold-standard laser for endocorporeal laser lithotripsy (ELL) because of its effectiveness and safety [1,2,3]. This long-pulsed infrared laser has a wavelength of 2100 nm, which is highly absorbed by water [1]. When we press the laser pedal, part of the initial laser energy is absorbed by the surrounding fluid, vaporizing the surrounding water until a vapor bubble is formed at the tip of the laser fiber [1,4]. This bubble expands outward and guides the laser pulse directly to the target; this is also known as the “Moses effect” (ME) [5,6,7]. In this way, the stone is heated and the chemical decomposition begins (photothermal mechanism). Simultaneously, the water contained within the pores of the stone surface also absorbs the laser energy and vaporizes, causing microexplosions and stone bursting from the inside (thermomechanical ablation) [8,9,10]. However, it was proposed that one half or more of the laser pulse is lost in the bubble formation [11]. Taking this assumption into consideration, the Moses idea was born in 1994 to allow a higher percentage of the laser pulse to reach the target. This idea modulated one pulse to have two sub-pulses, each with a different peak power. A short, low-energy initiation pulse would generate the vapor bubble, followed by a longer, more energetic and full-strength pulse after the full expansion of the vapor bubble [11]. It was not until 2017 that this idea became available in the urology market, known as Moses^TM^ technology (MT, Lumenis^®^). This laser developed by Lumenis is a 120 W holmium laser (Lumenis Pulse^TM^ 120 H; Lumenis, San Jose, CA, USA) that can reach up to 80 Hz. Recently, Lumenis released Moses 2.0, a new version of this high-power (HP) laser that can go up to 120 Hz. Nonetheless, is this type of technology really superior to the standard low-power (LP) Ho:YAG laser?

The aim of this narrative review is to provide an overview of the clinical experience with MT, including comparative studies, as well as an analysis of the first studies carried out with the recently launched Moses 2.0.

## 2. Methods

A literature review was carried out in June 2022 using the EMBASE and PubMed databases. An additional search was performed in the medical section of the publisher Mary Ann Liebert for peer-reviewed abstract presentations that were not indexed in the previously mentioned databases. All clinical experiences with Moses 1.0 and Moses 2.0 were included in the present study, including case reports and conference abstracts. Concerning Moses 2.0, all laboratory studies were selected. The inclusion criteria were very broad; we focused on all studies that treated ureteral and kidney stones endoscopically with the mentioned technology. Exclusion criteria included the use of MT in a non-endoscopic lithotripsy context and studies performed exclusively on pediatric patients. This review followed the Preferred Reporting Items for Systematic Reviews and Meta-Analyses (PRISMA) statement [10].

Different searches were performed with the following Medical Subject Heading (MeSH) terms and keywords: “holmium”, “laser”, “Moses”, “Lumenis”, “kidney”, “ureter”, “lithotripsy”, “endourology”, “stones”, and “lithiasis”. Boolean operators (AND, OR) were used to refine the search. We also reviewed the references for each included study. There was no time limit or language restriction.

Owing to the heterogeneity of the study outcomes and the lack of a standardized quality appraisal, a narrative synthesis rather than a quantified meta-analysis of the data was performed.

## 3. Results

**Figure 1:**
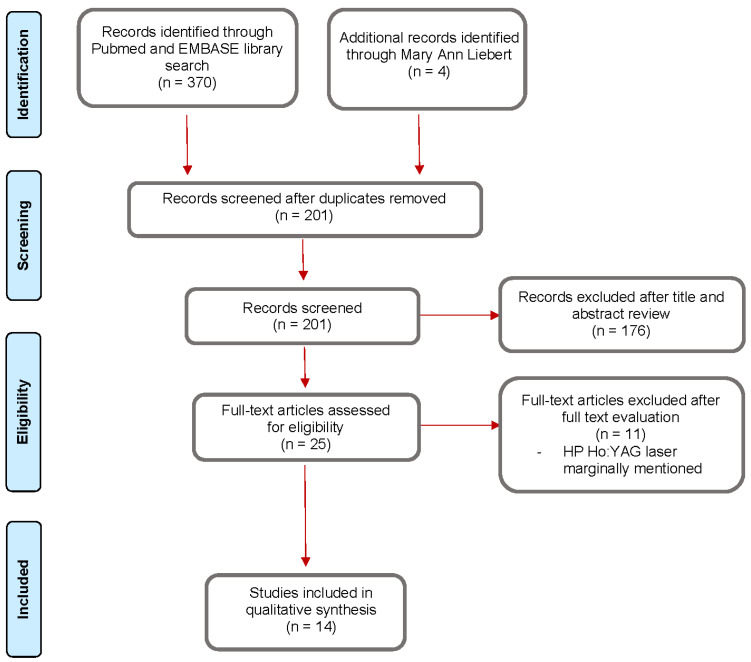
Flow chart of the literature review.

The PubMed and EMBASE search returned 370 articles and four additional abstracts were added after the Mary Ann Liebert search. After removing duplicates and reviewing the abstracts and texts collected, a total of 14 full-text articles and peer-reviewed abstract presentations were included for qualitative analysis: 11 clinical experiences (10 full-text clinical trials and one peer-reviewed abstract) with Moses 1.0 and Moses 2.0, and three laboratory studies (peer-reviewed abstracts) with Moses 2.0 only. The selection process is summarized in Figure 1.

Based on the purpose of this report, the clinical results obtained have been divided into three sections: Moses 1.0 clinical outcomes (Table 1), Moses 2.0 laboratory experiences (Table 2) and MT 2.0 clinical experiences (Table 3).

### 3.1. Moses 1.0 Clinical Outcomes

Clinical trials have been performed using this technology for ELL, including percutaneous nephrolithotomy (PCNL) and ureteroscopy (URS). A study by Leotsakos et al. [12] showed promising results when using MT in ultra-mini PCNL (UMPCNL). For stones of 7810.6 mm^3^, the laser was configured in Moses contact (MC) mode with the following settings: 0.6–0.8 J/80 Hz. After lasing for 13 min, 39.7 kJ were consumed. According to the authors, the coupled UMPCNL–MT could replace the standard PCNL for treating stones of any size. Another study [13] used both Moses modes, MC and Moses distance (MD) in mini-PCNL (stones < 3 cm); the laser settings were 0.4–0.6 J/40–60 Hz and the lasing time was 7.9 min. In both studies, the stone-free rate (SFR) was similar (91.7–100%).

In four URS studies, Moses 1.0 was compared to the regular mode of a HP Ho:YAG laser [14,15,16,17]. Most of the studies used 200 μm laser fibers [14]. The stone density was similar in all studies. All authors performed laser lithotripsy using low energy (0.3–0.4 J) and high frequencies (60–80 Hz) for dusting and inverse settings for fragmentation (0.8–1 J/8–10 Hz) in ureteral and renal stones. From two studies treating ureteral stones, one specified that most of them were fragmented and basketed [15], while the other one did not specify different laser settings when located in the ureter [17]. Knoedler et al. [17] divided the results based on the stone location and found that ureteral stones and kidney stones used similar lasing times in both Moses and regular mode. There was only one study that showed differences in the lasing time of the Moses mode vs. the regular mode [16], at 4.99 vs. 5.94 min, respectively.

In each comparative study, the total energy consumed did not statistically differ (3.8–6.4 kJ) [14,15,17]. From all the selected studies, Ibrahim et al. [15] used more energy in both the regular and Moses mode (11.1 vs. 10.8 kJ)—perhaps because of the greater stone size (1.4–1.7 cm). The SFR of the HP Ho:YAG lasers did not significantly differ between the two modes.

In two URS studies, Moses 1.0 was compared to the regular mode of a LP Ho:YAG laser [18,19]. The LP Ho:YAG lasers used in these studies were the Dornier Medilas^®^ H20 [18] and the Lumenis Holmium 20 W laser [19]. For similar-size stones (<1.5 cm), one study used laser fibers of different sizes (200, 365 and 550 μm) for both modes [18], and the other one used 200 μm laser fibers only [19]. Both studies used low-energy and high-frequency laser settings (0.4–0.8 J/63 or 20–25 Hz) in the Moses mode, whereas low-energy and frequency settings (0.4–0.8 J/12–18 Hz) were used in the LP group [18,19]. For ureteral stones, neither study specified a change in the mentioned settings. Statistical differences were seen in the lasing time, total energy, ablation speed (mm^3^/s) and ablation efficiency (J/mm^3^). The lasing time was two-times greater in the regular group compared to the Moses group (6.6 vs. 3.3 min) [18]. The Moses group used a higher total energy (4.7 vs. 3.6 kJ) and more J/mm^3^ (17.2 vs. 13 J/mm^3^) [18].

Concerning the ablation speed (mm^3^/s), one study described it as s/mm^3^. We assume that the authors wanted to report that the ablation speed of the Moses mode was faster than that of the regular mode [18]. Both groups had similar SFRs [18,19].

### 3.2. Moses 2.0 Laboratory Experiences

As Moses 2.0 has just recently entered the market, there is not much information about it. Laboratory experiences with this new mode have been described in three abstracts [20,21,22] coming from the same group of researchers. Two of them compared the MD with the new Moses 2.0, with an extended frequency rate (EFR) [20,22]. The laser settings were similar for the MD mode (0.2–0.4 J and 80 Hz) and for the Moses 2.0 mode (0.2–0.3 J and 100–120 Hz). One of the two studies [20] showed that at a stone distance of 0 mm, the EFR had a superior ablation volume to the MD across all pulse energies—especially in soft stones and when using low-energy settings. With low pulse energies and higher pulse frequencies, Moses 2.0 was more efficient because of its lower retropulsion. The other study showed that a slower movement of the fiber over the stone resulted in a greater stone ablation, but the volumetric stone treatment decreased as stone damage plateaued after repeated pulses to the same area [22]. In addition, in terms of pulse energy, 0.3 J was superior to 0.2 J at all frequencies. The same group compared the Moses 2.0 with the MD and short pulse (SP) modes in soft and hard stones [21]. For the ablation of hard stones, EFR 0.5 J/100 Hz was superior to SP 0.5 J/70 Hz; EFR 0.5 J/90 Hz was superior to SP 0.5 J/70 Hz and MD 0.5 J/70 Hz. EFR 0.5 J/90 Hz resulted in a higher fragmentation rate. On soft stones, SP 0.6 J/80 Hz left fewer residual fragments > 2 mm than SP 0.5 J/70 Hz, MD 0.5 J/70 Hz and EFR 0.5 J/100 Hz. At similar energy settings, EFR did not demonstrate benefits on soft stone ablation. The 1 J/20 Hz SP and MD performed worst on soft and hard stones. According to the authors, the EFR settings enabled efficient, high-power popcorn lithotripsy; the most effective popcorn lithotripsy in any stone type was achieved with both EFR and SP modes.

### 3.3. MT 2.0 Clinical Experiences

There is limited clinical experience with Moses 2.0. We only found three reports, one of which was a single case report [23,24,25]. All studies performed ELL in kidney stones and used 230 μm laser fibers. Khajeh et al. reported a single case with this new Moses mode in a patient with a 1.7 cm renal stone [23]. To debulk the stone, 0.3 J/120 Hz contact laser lithotripsy was used. After fragmenting the stone, the Moses mode was changed to non-contact pop-dusting lithotripsy. The lasing time was 23 min, and the total energy used was 33.3 kJ. Only a 1 mm residual fragment remained after 6 weeks. Researchers found that 0.3 J/120 Hz is the optimal setting for both non-contact and contact laser lithotripsy.

Majdalany et al. [24] compared Moses 1.0 vs. Moses 2.0 in 29 patients. For Moses 1.0, the laser settings were 0.5 J/50–80 Hz and for Moses 2.0, the settings were 0.5 J/50–120 Hz for similar stones. The Moses 1.0 group had a shorter lasing time and consumed almost two-times less energy than the Moses 2.0 group. A faster ablation speed was observed in the Moses 2.0 group, whereas a lower J/mm^3^ was observed in the Moses 1.0 group. However, a higher SFR was seen in the Moses 2.0 group (90% vs. 71%).

Finally, Rezakahn et al. [25] performed a study explaining their first experience with Moses 2.0 for stones of around 1 cm. The contact laser lithotripsy used 0.2–0.3 J/100–120 Hz for debulking, and the MD mode with 0.5 J/80 Hz was used once the stone was fragmented. The SFR, lasing time and total energy used were similar to the previous study by Majdalany et al. [24].

## 4. Discussion

With Moses technology, the ME can be controlled by dividing a laser pulse into two sub-pulses [11]. Moses 1.0 is capable of working with two pulse modulations: MC, when working at a distance of 1 mm, and MD, when working at a distance of 2 mm [26]. MT uses laser fibers of different sizes; MOSES™ D/F/L is available in 200, 365 and 550 µm [27]. There were great expectations for this technology when it was released in 2017, due to what had been seen in lab tests, such as a more efficient ablation of soft stones when used in MC mode [28]. Moreover, it was shown that increasing the fiber speed increased the stone ablation when using high-frequency settings (20 to 60 Hz) [29].

Looking at clinical experiences in real life, we see different outcomes. In URS, there was no difference in lasing time between Moses 1.0 and HP Ho:YAG lasers in all clinical trials [14,15,17], except one [16]. In that study [16], Moses 1.0 had a shorter lasing time by only about 1 min. However, Moses 1.0 showed a significantly shorter lasing time and operative time when compared with LP Ho:YAG lasers (20 W) [18,19]. All studies had similar SFRs. These results are consistent with those published by Ventimiglia et al. [30]; they performed a systematic review and meta-analysis comparing LP vs. HP Ho:YAG lasers for ELL and they found that HP lasers had similar stone-free and complication rates than LP ones, but a shorter operative time. This advantage was lost when the stone burden was taken into account. In this study [30], the HP group had smaller stones—about two-times smaller than the LP group. In our review, it was noted that Moses 1.0 reduced the lasering time by half [18], but it was also noted that Moses 1.0 consumed more energy than the regular LP Ho:YAG mode. In Moses 1.0, the frequency was up to five times higher, resulting in greater energy consumption [18]. This brings us to the laser efficacy definition, which is the total amount of energy needed to ablate 1 mm^3^ of a stone (J/mm^3^) [31]. It should be noticed that all LP lasers included in this review work at 20 W. Nowadays, we can find 30–35 W LP Ho: YAG lasers, reaching up to 30 Hz. It has previously been described that the laser efficacy of the average LP Ho:YAG laser (35 W) is 19 J/mm^3^ [31] and that they are as effective as HP ones [32]. This study found that the Moses 1.0 consumed 17.2 J/mm^3^, which is in line with what the LP lasers consume [18], meaning that Moses 1.0 and LP lasers (35 W) have a similar efficacy.

Furthermore, the overall energy consumption was similar across all comparative studies (Moses 1.0 vs. HP Ho:YAG lasers), ranging from almost 4 kJ for stones of 1.2 cm to almost 11 kJ for stones of almost 1.5 cm [14,15,17]. HP lasers have been related to dangerous increases in water temperature inside the kidney [30,33,34,35,36]. Despite the fact that this effect can be mitigated by high-flow irrigation, it is not uncommon for forced irrigation during ELL to cause pyelovenous backflow due to increases in intrarenal pressures (i.e., 300 mmHg) [37]. As mentioned, all comparative studies included in this review showed no significant difference in SFR. This raises the question of whether HP Ho:YAG lasers, including those with pulse modulation (MT: Moses 1.0), are actually more efficient. Despite having new in vitro publications on pulse modulation with MT in laser lithotripsy [38], this subject has not yet been clarified in clinical experience. According to a recent review of Ho:YAG lasers with MT, the clinically significant evidence required to change daily practice is still lacking [39]. Aside from this, in terms of cost analysis, not enough savings are provided to offset the higher cost of the fibers and software [40].

On the other hand, the use of high frequencies in MT lithotripsy also has an impact on the morpho-constitutional analysis of the stone. Keller et al. [41] performed an analysis of the disintegration products from Holmium lithotripsy for the most frequently encountered crystalline constituents of urinary stones. They showed that MT seems to produce a more pronounced disruption of morphological characteristics, suggesting that the photothermal effect may also play a role in the resulting stone dust—which may not adequately reflect the initial crystalline organization of the stones before laser lithotripsy.

Comparative trials between MT and the new thulium fiber laser (TFL) are also lacking. There is one lab study that compared the MT with the TFL in terms of pulse shape, stone retropulsion and ablation efficiency at equivalent laser settings [42]. For HP Ho:YAG lasers, the modalities were short pulse (SP), long pulse (LoP) and Moses pulse (MP), and for TFL, the modalities were regular pulse (RP) and dual pulse (DP). Based on the results, the authors concluded that MP and LoP had higher retropulsion than TFL, while TFL had higher ablation volumes than the Ho:YAG laser.

Additionally, a new version of the MT was released in 2020—Moses 2.0. Moses 2.0 includes a new pulse modality called “optimized Moses”, a choice that appears when the user wants to use an extended frequency rate (EFR) of 80–120 Hz [43]. Lab studies have shown that when comparing MD and Moses 2.0 with EFR, EFR had a superior stone ablation volume than MD at SD 0 mm [20]. Nonetheless, this advantage was lost when stone density was taking into consideration, showing a more efficient popcorn lithotripsy and a higher fragmentation rate on hard stones only [21]. There was also higher ablation volume and less retropulsion with lower pulse energies, as well as greater laser efficiency with higher pulse frequencies [20]. A further observation is that the ablation speed and laser efficiency were higher with a higher scanning speed of the laser fiber [22]. However, whether this can be replicated in real life remains unanswered, since rapid scanning must be limited to safely maneuver the ureteroscope while maintaining close contact with the stone [22].

In terms of clinical experience, there are only two clinical trials and one single case report. Majdalany et al. [24] compared Moses 1.0 vs. Moses 2.0 in stones of similar size and density. Patients were treated both with contact and non-contact (pop-dusting) lithotripsy, with laser settings of 0.2–0.3 J and 50 Hz (Moses 1.0) to 120 Hz (Moses 2.0) during the contact phase, and 0.5 J and 50–80 Hz during the non-contact phase. Moses 1.0 had better laser efficacy (32.4 vs. 47.8 J/mm^3^) and SFR, but it also required double the energy to ablate 1 mm^3^ of stone volume compared to the LP Ho:YAG laser [31]. Moses 2.0 ablation speed was slightly faster (0.97 vs. 0.88 mm^3^/s) but had a longer operative time. Rezakahn et al. [25] obtained similar results with the Moses 2.0 only in terms of lasing time and total energy consumed.

This review is not without limitations. First, due to the lack of comparative studies between MT (1.0 and 2.0) and LP/HP Ho:YAG lasers, the level of evidence is limited. Second, few studies have considered in the ablation speed and laser efficacy, which are important parameters to evaluate lasers’ efficiency accurately.

## 5. Conclusions

Currently, there are no high-quality studies that support the use of HP lasers with MT over other lasers such as LP Ho:YAG lasers or TFL lasers. It seems that LP Ho:YAG lasers are still a good alternative for ELL. Further comprehensive experimental studies and clinical trials comparing MT with the new TFL are required.

## Figures and Tables

**Table 1:** Clinical experience with Moses 1.0. I: Intervention, *n*: sample size, SD: Standard deviation, RCT: Randomized control trial, P: Prospective, R: Retrospective, RC: Retrospective comparative, PC: Prospective comparative, RM: Regular mode, MC: Moses contact, MD: Moses distance, D: Dusting, F: Fragmentation, SFR: stone-free rate, N.A: Not available, *: statistically significant difference (*p* < 0.05), **: mostly fragmented ureteral stones.

Author, Year, Type of Study	I	*n*	Type of Laser	Pulse Modulation Setting: *n*	Fiber Size (μ)	Laser Settings	Stone Size Mean ± SD (cm)	Stone Volume Mean ± SD (mm^3^)	Stone Density Mean ± SD (HU)	Stone Location	Lasing Time Mean ± SD (min)	Operative Time Mean ± SD (min)	Energy Mean ± SD (kJ)	Ablation Speed (mm^3^/s)	J/mm^3^	SFR (%)
Leotsakos et al., 2020, R [11]	Ultra-mini PCNL	12	Lumenis Moses Pulse^TM^ 120 H	MC	550	0.6–0.8 J/80 Hz	3.15	7810.6	1252	Kidney	13 ± 16	86.4 ± 36.8	39.7 ± 52	N.A	N.A	91.7
Reddy et al., 2021, P [12]	Ultra-mini PCNL	110	Lumenis Moses Pulse^TM^ 120 H	MC and MD	365	0.4–0.6 J/40–60 Hz	1.75	N.A	1140	Kidney	7.9	38.6	N.A	N.A	N.A	100
**Moses technology vs. High-power Ho:YAG laser regular mode**																
Mullerad et al., 2017, P [13]	URS	34	Lumenis Moses Pulse^TM^ 120 H	Moses: 23	200, 365, 550	N.A	N.A	781.9 median	901.5	Kidney or ureter	6 (median)	N.A	4.5 (median)	N.A	N.A	N.A
				RM: 11	200, 365			422.5 median	867		10 (median)		6.4 (median)			
Ibrahim et al., 2020, RCT [14]	URS	72	Lumenis Moses Pulse^TM^ 120 H	Moses: 36	200	D: 0.4 J/80 Hz	1.7 ± 1.5	N.A	991 ± 213	Kidney or ureter **	6.1 ± 9.8	41.1 ± 21.1 *	10.8 ± 14.1	N.A	N.A	88.4
				RM: 36		F: 1 J/10 Hz	1.4 ± 0.97		841 ± 348		7.4 ± 5.7	50.9 ± 27.9 *	11.1 ± 20.3			88.3
Wang et al., 2021, RC [15]	URS	216	Lumenis Moses Pulse^TM^ 120 H	MC: 114	200	0.3 J/60 Hz	1.2	674 ± 41	990 ± 150	Kidney	4.99 ± 1.06 *	18.39 ± 5.13 *	N.A	N.A	N.A	86.8
				RM: 102			1.2	683 ± 39	994 ± 150		5.94 ± 0.96 *	21.17 ± 6.78 *				85.3
Knoedler et al., 2022, RC [16]	URS	176	Lumenis Moses Pulse^TM^ 120 H	Moses: 110	200	D: 0.3 J/80 Hz	1.18 ± 0.79	N.A	N.A	Kidney and/or ureter	7.5 ± 11.1	43.5 ± 32.1	5.1 ± 6.7	N.A	N.A	52.3
				RM: 66		F: 0.8 J/8 Hz	1.16 ± 0.92				6.7 ± 7.9	39.8 ± 24.6	3.8 ± 4.8			65.3
**Moses technology vs. Low-power Ho:YAG laser regular mode**																
Mekayten et al., 2019, RC [17]	URS	631	Lumenis Moses Pulse^TM^ 120 H	Moses: 169	200, 365, 550	0.5 J/63 Hz * (mean)	N.A	427	1085 *	Kidney or ureter	3.3 *	21.13 *	4.7 ± 5 *	0.8 *	17.2 *	84.5
			Ho:YAG 20 W	RM: 462		0.7 J/14 Hz * (mean)		367	1022 *		6.6 *	31.84 *	3.6 ± 4 *	1.51 *	13 *	87.2
Pietropaolo et al., 2021, RC [18]	URS	76	Lumenis Moses 60 W	Moses: 38	200	0.4–0.8 J/20–25 Hz	1.09 ± 0.4	N.A	N.A	Kidney or ureter	N.A	51.6 ± 17.1 *	N.A	N.A	N.A	100
			Ho:YAG 20 W	RM: 38		0.4–0.8 J/12–18 Hz	1.18 ± 0.4					82.1 ± 27.0 *				97.3

**Table 2:** Laboratory experience with Moses 2.0.

	MD Settings	SP Settings	Moses 2.0 (EFR) Settings	Results
Whelan, P., et al., 2021, abstract [19]	0.3 J/80 Hz (24 W);	-	0.2 J/120 Hz (24 W);	At comparable power settings, EFR had a superior stone ablation volume than MD at SD 0 mm.
				***Ablation volume***: Higher with lower energy in soft stones.
	0.4 J/80 Hz (32 W)		0.3 J/120 Hz (36 W)	***Retropulsion***: Lower with lower pulse energy.
				***Efficiency***: Higher with higher pulse frequency
Whelan, P., et al., 2021, abstract [20]	0.5 J/70 Hz (35 W)	0.5 J/70 Hz (35 W)	0.3 J/120 Hz (36 W)	Hard stones:
	0.6 J/80 Hz (48 W)	0.6 J/80 Hz (48 W)	0.4 J/100 Hz (40 W)	- EFR: Provides a more efficient popcorn lithotripsy.
	1 J/20 Hz (20 W)	1 J/20 Hz (20 W)	0.5 J/100 Hz (50 W)	- EFR 0.5 J/90 Hz offers the greatest fragmentation among the other modes.
			0.5 J/90 Hz (45 W)	Soft stones: EFR did not demonstrate benefits over the other modes, for a similar total power.
Whelan, P., et al., 2021, abstract [21]	0.3 J/80 Hz (24 W)	-	0.2 J/120 Hz (24 W)	***Ablation speed***: Is highest, with higher scanning speeds of the laser fiber.
			0.2 J/100 Hz (20 W)	0.3 J is superior to 0.2 J at all frequencies
	0.2 J/80 Hz (16 W)		0.3 J/120 Hz (36 W)	
			0.3 J/100 Hz (30 W)	***Efficiency***: Higher with a more rapid scanning of the laser fiber

**Table 3:** Clinical experience with Moses technology. I: Intervention, *n*: sample size, SD: Standard deviation, SC: Single case report, P: Prospective, R: Retrospective, RC: Retrospective comparative, MD: Moses distance, SFR: stone-free rate.

Author, Year, Type of Study	I	*n*	Type of Laser	Pulse Modulation Setting: *n*	Fiber Size (μ)	Laser Settings	Stone Size Mean ± SD (cm)	Stone Volume Mean ± SD (mm^3^)	Stone Density Mean ± SD (HU)	Stone Location	Lasing Time Mean ± SD (min)	Operative Time Mean ± SD (min)	Energy Mean ± SD (kJ)	Ablation Speed (mm^3^/s)	J/mm^3^	SFR (%)
Khajeh et al., 2021, SCR [22]	URS	1	Lumenis Moses Pulse^TM^ 120 H	Moses 2.0	230	Debulk:	1.7	N.A	N.A	Kidney	16	23	33.3	N.A	N.A	N.A
						0.3 J/120 Hz										
						Pop-dusting:										
						0.5 J/80 Hz										
Majdalany et al., 2021, RC [23]	URS	29	Lumenis Moses Pulse^TM^ 120 H	Moses 1.0: 18	230	0.5 J/50–80 Hz	0.94	242	784	Kidney	5.3	10.4	6.4	0.88	32.4	71
				Moses 2.0: 11		0.5 J/50–120 Hz		368	865		7.0	14.3	12.4	0.97	47.8	90
Rezakahn et al., 2021, P [24]	URS	12	Lumenis Moses Pulse^TM^ 120 H	Moses 2.0	230	Debulk:	1.04	N.A	865	Kidney	6.9	15	12	N.A	N.A	82
						0.2–0.3 J/100–120 Hz										
						Pop-dusting:										
						0.5 J/80 Hz (MD)										

## Data Availability

Not applicable.
